# The Tenth International Medical Congress

**Published:** 1890-04

**Authors:** 


### Society Meetings.

### THE TENTH INTERNATIONAL MEDICAL CONGRESS.

110 West 34th Street,
NEW YORK, March 7, 1890.

*Editors Buffalo Medical and Surgical Journal:*

I am directed by the Secretary-General of the Tenth International Congress to give the greatest possible publicity to the circular, the main points of which I herewith transmit to you, with the request that they be published.

Very respectfully yours,
A. JACOBI, M. D.

INVITATION FOR AN INTERNATIONAL MEDICAL AND SCIENTIFIC EXHIBITION.

In connection with the Tenth International Medical Congress, to be held in Berlin, between the 4th and 10th of August, 1890, there is to be an International Medical and Scientific Exhibition. The exhibits will be of an exclusively scientific nature, as follows:

New or improved scientific instruments and apparatuses for biological and strictly medical purposes, inclusive of apparatuses for photography and spectral analysis as far as applicable to medicine.

New objects and preparations in pharmacological chemistry and pharmacy.

New foods.

New or improved instruments subservient to any of the departments of medicine, including electrotherapy.

New plans and models for hospitals, convalescent homes, and disinfecting and bathing institutions and apparatuses.

New arrangements for nursing, including transportation, baths, etc.

New apparatuses in hygiene.

Applications or inquiries inscribed "Ausstellungs-Angelegenheit," and accompanied with a printed card containing the name and address of the firm thus applying, ought to be directed to the Secretary-General, Dr. O. Lassar, Carlstrasse, No. 19, Berlin, N. W., Germany.

R. VIRCHOW, President.
E. VON BERGMANN, E. LEYDEN, W. WALDEYER, Vice-Presidents.
O. LASSAR, Secretary-General.

